# Influence of Peroxisome Proliferator-Activated Receptor (PPAR)-gamma Coactivator (PGC)-1 alpha gene rs8192678 polymorphism by gender on different health-related parameters in healthy young adults

**DOI:** 10.3389/fphys.2022.885185

**Published:** 2022-07-22

**Authors:** Adrián Montes-de-Oca-García, Juan Corral-Pérez, Daniel Velázquez-Díaz, Alejandro Perez-Bey, María Rebollo-Ramos, Alberto Marín-Galindo, Félix Gómez-Gallego, Maria Calderon-Dominguez, Cristina Casals, Jesús G. Ponce-González

**Affiliations:** ^1^ MOVE-IT Research Group, Department of Physical Education, Faculty of Education Sciences, University of Cadiz, Cádiz, Spain; ^2^ Research Unit, Biomedical Research and Innovation Institute of Cadiz (INiBICA), Puerta Del Mar University Hospital, University of Cadiz, Cádiz, Spain; ^3^ Department of Psychology, Brain Aging and Cognitive Health Laboratory, University of Pittsburgh, Pittsburgh, PA, United States; ^4^ Department of Physical Education, GALENO Research Group, Faculty of Education Sciences, University of Cadiz, Cádiz, Spain; ^5^ Faculty of Health Sciences, International University of La Rioja, Logroño, Spain; ^6^ Biomedicine, Biotechnology and Public Health Department, University of Cadiz, Cádiz, Spain

**Keywords:** genetic association studies, cardiometabolic risk, metabolism, healthy lifestyle, PPARGC1A, caucasian, obesity, physical exercise

## Abstract

This study aimed to analyze the influence of the peroxisome proliferator-activated receptor (PPAR)-gamma coactivator (PGC)-1 alpha (PPARGC1A) gene rs8192678 C>T polymorphism on different health-related parameters in male and female young adults. The PPARGC1A gene rs8192678 polymorphism was ascertained by polymerase chain reaction in 74 healthy adults (28 women; 22.72 ± 4.40 years) from Andalusia (Spain). Health-related variables included cardiometabolic risk, anthropometry and body composition, biochemical parameters, insulin sensitivity (QUICKI and HOMA-IR indexes), blood pressure (BP) at rest and after exercise, diet, basal metabolism, physical activity, maximal fat oxidation, and cardiorespiratory fitness. Our results showed differences by PPARGC1A gene rs8192678 C>T polymorphism in body mass (*p* = 0.002), body mass index (*p* = 0.024), lean body mass (*p* = 0.024), body fat (*p* = 0.032), waist circumference (*p* = 0.020), and BP recovery ratio (*p* < 0.001). The recessive model (CC vs. CT/TT) showed similar results but also with differences in basal metabolism (*p* = 0.045) and total energy expenditure (*p* = 0.024). A genotype*sex interaction was found in the QUICKI index (*p* = 0.016), with differences between CC and CT/TT in men (*p* = 0.049) and between men and women inside the CT/TT group (*p* = 0.049). Thus, the PPARGC1A gene rs8192678 C>T polymorphism is associated with body composition, basal metabolism, total energy expenditure, and BP recovery, where the CC genotype confers a protective effect. Moreover, our study highlighted sexual dimorphism in the influence of PPARGC1A gene rs8192678 C>T polymorphism on the QUICKI index.

## Introduction

The peroxisome proliferator-activated receptor-gamma (PPARγ) coactivator-1 alpha (PPARGC1A, also known as PGC-1α), initially identified as a coactivator of PPARγ, is a transcriptional coactivator of the PPAR superfamily of nuclear receptors (Zhu et al., 2009). It interacts with PPARγ, influencing many other transcriptional factors that may affect health status (Janani and Ranjitha Kumari, 2015). PGC-1α is involved in mitochondrial biogenesis, glucose utilization, gluconeogenesis, insulin signaling, fatty acid oxidation, and thermogenesis (Petr et al., 2018). In humans, the PPARGC1A gene coactivates multiple transcriptional factors involved in a wide variety of biological responses (Tharabenjasin et al., 2019); thus, it is a crucial protein in human metabolism (Liang and Ward, 2006).

The three most studied Single Nucleotide Polymorphisms (SNPs) of the PPARGC1A gene are PPARGC1A rs8192678, PPARGC1A rs2970847, and PPARGC1A rs3736265. Regarding rs8192678 polymorphism, a C→T substitution leading to the change of glycine with serine in codon 482 (Gly482Ser), can be considered the most important polymorphism of PPARGC1A (Csép et al., 2017), with controversial results about the influence on gene expression (Csép et al., 2017) and, hence, on PGC-1α functions (Wu et al., 2016). In this line, the T allele (482Ser) of the PPARGC1A polymorphism has been associated with reduced expression of PPARGC1A (Wu et al., 2016).

The PPARGC1A gene rs8192678 polymorphism has been previously related to the development of obesity (Mirzaei et al., 2012; Franks et al., 2014; Costa-Urrutia et al., 2018; Zamaninour et al., 2018), biochemical parameters such as glucose, insulin, triglycerides and inflammatory markers, resting energy expenditure (Mirzaei et al., 2012), and physical fitness level (Costa-Urrutia et al., 2018). In fact, it has been speculated that altered PPARGC1A gene expression might contribute to developing insulin resistance by impaired metabolic pathways related to cardiovascular risk (e.g., PPAR-mediated adipocyte differentiation, lipid oxidation, gluconeogenesis in the liver, or glucose transport in the muscles) (Vohl et al., 2005). Furthermore, PPARGC1A rs8192678 polymorphism could affect muscle fiber type composition due to the influence on mitochondrial biogenesis through PGC-1α (Petr et al., 2018; Tharabenjasin et al., 2019), which is related to fat oxidation capacity (Wu et al., 2016). However, the influence of PPARGC1A (Gly482Ser) polymorphism on fat oxidation capacity during exercise remains unknown despite its relevance as an indicator of metabolic flexibility (Goodpaster and Sparks, 2017).

Notwithstanding, the results of previous studies about the PPARGC1A gene rs8192678 C>T polymorphism and its association with health are not always uniform, being sometimes contradictory or insufficient. Thus, this study aimed to investigate the influence of PPARGC1A gene rs8192678 C>T polymorphism on different health-related parameters in male and female young adults by including body composition, cardiorespiratory fitness (VO_2_max), basal metabolism, total energy expenditure, maximal fat oxidation (MFO), physical activity, diet, blood pressure, biochemical parameters, insulin sensitivity, and cardiometabolic risk (CMR).

## Materials and methods

### Design

This study, with a cross-sectional design, was part of the “NutAF research Project” (Rebollo-Ramos et al., 2019; Montes-de-Oca-García et al., 2020; Corral-Pérez et al., 2021; Montes-de-Oca-García et al., 2021). The PPARGC1A gene rs8192678 C>T polymorphism was assessed in a young adult population, investigating any feasible relation with various health outcomes. The Ethical Committee of the Hospital Puerta del Mar (Cadiz, Spain), according to the Helsinki Declaration, approved this study. The participants were fully informed of the aims of the study and any possible side effect, all of them gave their written informed consent. The tests were conducted at the University of Cadiz (Spain), specifically in the Laboratory of Physical Activity and Exercise.

### Subjects

A total of 74 Spanish Caucasian subjects [28 women; 22.72 ± 4.40 years old; 25.81 ± 5.61 kg m^−2^ of body mass index (BMI)] were enrolled. According to one of our main variables, BMI, and using the G*Power software (v. 3.1.9.6, University of Kiel, Germany), we obtained a compute achieved power of 0.95, and an effect size of 0.8, with our sample size. All participants were young adults with different weight status, but healthy (without diseases), living in the province of Cadiz located in Andalusia (Spain). The Table 1 includes the participants’ characteristics. The criteria of inclusion comprised being between 18 and 45 years old, maintaining a stable body mass (±2 kg) in the last 6 months and BMI between 20 and 40 kg · m^−2^. Participants were excluded from the study if the met the following criteria: having changed their usual diet during the last 6 months (e.g., a weight-loss diet), being an active smoker, or having any cardiovascular disease, injury or any other condition that prevented physical activity.

**TABLE 1:** Participants’ characteristics and differences between sexes.

	*n*	Total(*n* = 74)	Men(*n* = 46)	Women(*n* = 28)	*p*-value	*d*
Age (years)	74	22.6 ± 4.2	22.2 ± 3.6	23.2 ± 5.1	0.397	−0.21
Height (cm)	74	172.1 ± 8.7	176.7 ± 6.1	164.6 ± 6.8	**<0.001**	**1.87**
Body Mass (kg)	74	76.3 ± 15.8	78.7 ± 14.8	72.3 ± 16.9	0.089	0.41
Body Mass Index (kg · m^−2^)	74	25.8 ± 5.6	25.1 ± 4.2	26.8 ± 7.4	0.273	−0.28
Lean Body Mass (kg)	74	54.4 ± 8.8	59.4 ± 6.5	46.1 ± 4.8	**<0.001**	**2.34**
Body fat (kg)	74	18.7 ± 11.3	15.8 ± 9.2	23.3 ± 12.9	**0.010**	−**0.67**
Waist circumference (cm)	68	83.6 ± 14.7	85.2 ± 13.2	81.4 ± 16.6	0.298	0.25
VO_2_max (mL · kg^−1^ · min^−1^)	73	40.9 ± 11.9	45.5 ± 10.5	33.7 ± 10.3	**<0.001**	**1.13**
Basal metabolism (kcal · min^−1^)	73	1.3 ± 0.2	1.4 ± 0.2	1.1 ± 0.1	**<0.001**	**1.71**
Total energy expenditure (kcal/day)	67	2,377 ± 411	2,554 ± 364	2,078 ± 299	**<0.001**	**1.43**
Dietary energy intake (kcal/day)	73	2,536 ± 686	2,724 ± 672	2,233 ± 604	**0.002**	**0.77**
Energy balance (kcal/day)	66	166 ± 725	190 ± 755	126 ± 685	0.730	0.09
Resting Fat Oxidation (mg · min^−1^)	73	99 ± 28	103 ± 30	94 ± 22	0.181	0.34
Absolute MFO (mg · min^−1^)	73	375 ± 155	406 ± 171	325 ± 111	**0.016**	**0.56**
R-MFO [mg (kg · m^−2^)^−1^ min^−1^]	73	6.9 ± 2.8	6.3 ± 2.6	7.8 ± 2.9	**0.040**	−**0.54**
Total MVPA (min/week)	67	371 ± 134	369 ± 118	375 ± 159	0.850	−0.05
MD adherence (0–14 range)	73	6.9 ± 1.7	7.2 ± 1.9	6.5 ± 1.4	0.132	0.38
Systolic blood pressure (mmHg)	73	114.8 ± 10.2	117.7 ± 7.9	110.1 ± 11.9	**0.002**	**0.75**
Diastolic blood pressure (mmHg)	73	68.9 ± 9.7	67.6 ± 9.1	71.1 ± 10.5	0.140	−0.35
Blood pressure recovery (ratio)	68	0.92 ± 0.06	0.93 ± 0.06	0.91 ± 0.07	0.190	0.31
Fasting glucose (mg · dl^−1^)	74	101.1 ± 10.2	103.4 ± 9.6	97.1 ± 9.9	**0.008**	**0.66**
Fasting insulin (ng · ml^−1^)	74	0.8 ± 0.7	0.8 ± 0.7	0.7 ± 0.6	0.860	0.04
HOMA-IR index	74	5.8 ± 5.6	6.1 ± 5.9	5.4 ± 4.9	0.673	0.10
QUICKI index	74	0.31 ± 0.02	0.30 ± 0.02	0.31 ± 0.02	0.616	−0.50
Fasting triglycerides (mg · dl^−1^)	74	69.8 ± 24.7	71.7 ± 24.9	66.8 ± 24.6	0.414	0.20
Tumor necrosis factor-α (ng · ml^−1^)	71	8.3 ± 7.6	9.4 ± 8.4	6.6 ± 5.7	0.121	0.39
Interleuking-6 (ng · ml^−1^)	55	0.4 ± 0.3	0.5 ± 0.3	0.4 ± 0.2	0.688	0.12
Leptin (ng · ml^−1^)	70	3.9 ± 4.5	1.9 ± 2.8	7.3 ± 4.9	**<0.001**	−**1.34**
Clustered CMR (Z-score)	67	0.5 ± 3.3	0.7 ± 3.2	0.1 ± 3.3	0.405	0.21
INFLAM-Clustered CMR (Z-score)	47	0.1 ± 3.1	0.2 ± 3.3	0.1 ± 2.3	0.891	0.04

Values are presented as mean ± standard deviation. Differences by sex appear in bold (*p* < 0.05 in the Student *t*-test). Abbreviations: *d*, Cohen’s d; MVPA, Moderate to vigorous physical activity; MD, Mediterranean diet; VO_2_max, Maximal oxygen uptake; R-MFO, Maximal fat oxidation rate relativized to legs lean mass divided by the height squared; HOMA-IR, Homeostasis Model Assessment of Insulin Resistance; QUICKI, QUantitative Insulin sensitivity cheCK Index; CMR, Cardiometabolic risk; INFLAM-Clustered CMR, Cluster of CMR including IL-6 and TNF-α.

### Procedures

The week before measurements, both diet and physical activity were registered asking to the participants to maintain their usual lifestyles. The day before measurements, participants were directed to maintain their habitual diet/hydration, to avoid alcohol and caffeine intake, and to prevent vigorous physical activity. At the laboratory, basal heart rate and blood pressure were measured, then fasting blood samples were obtained to assess the PPARGC1A gene rs8192678 C>T polymorphism and plasma markers including glucose, triglycerides, interleukin-6 (IL-6), tumor necrosis factor-α (TNF-α), insulin, and leptin. After that, participants underwent anthropometric and body composition assessments and, finally, the physical exercise tests to estimate MFO and maximal oxygen consumption (VO_2_max).

### Physical activity

Accelerometry was used to estimate physical activity (GT3X+, Actigraph TM, LLC, Fort Walton Beach, FL, United States) during the 7 days prior to laboratory tests. During waking hours, the accelerometer was worn on the hip, as previously described (Corral-Pérez et al., 2021). Measures were included in the analysis if at least 10 h/day and 4 days (with one weekend day) were registered. Finally, Moderate to Vigorous Physical Activity (MVPA) was estimated as any activity over 2,691 counts/min (Corral-Pérez et al., 2021). Light Physical Activity (150–2,690 counts/min) was also measured in order to estimate total energy expenditure (Corral-Pérez et al., 2021), which was calculated by adding the basal metabolism and the caloric expenditure of physical activity measured through accelerometry, taking into account the 10% more energy expenditure from the diet-induced thermogenesis.

### Dietary assessment

The week before laboratory measurements, all participants completed a dietary record by weighting for five consecutive days, including two weekend days. In order to quantify energy intakes (kcal per day), dietary records were analyzed by using the DIAL software (version 1.19). Likewise, the daily energy balance was calculated as the dietary energy intake minus the total energy expenditure. Concerning dietary patterns, the degree of adherence to the Mediterranean diet of the participants was measured through a 14-item questionnaire (García-Conesa et al., 2020).

### Heart rate and blood pressure

Systolic blood pressure (SBP) and diastolic blood pressure (DBP) were measured, after 10 min of sitting rest, by using an Omron M3 digital monitor (HEM-7051-E, Kyoto, Japan) as previously described (Montes-de-Oca-García et al., 2021). Heart rate was recorded at resting and during the exercise tests by using a Polar Team 2 monitoring equipment (Polar Electro Inc., Lake Success, NY, United States). Additionally, the blood pressure recovery from a maximal exercise test was determined by using the SBP in a third minute of recovery to peak exercise SBP ratio (Michaelides et al., 2013).

### Blood extraction

Fasting blood samples were taken from the antecubital vein and collected in EDTA tubes, one tube was stored as total blood for genomic typing and the second tube was centrifuged (2,500 rpm, 15 min, 4°C) to obtain plasma that was stored at −80 °C until analyses.

### Genomic typing of the PPARGC1A gene rs8192678 C>T polymorphism

Genomic Deoxyribonucleic acid (DNA) was extracted from peripheral blood anticoagulated with EDTA using a standard phenol/chloroform procedure followed by alcohol precipitation. Allelic discrimination analysis was performed by predesigned Life Technologies TaqMan^®^ SNP Genotyping Assays on demand for the PPARGC1A rs8192678 polymorphism (ID: C_1643192_20). A quantitative Real-Time polymerase chain reaction (qRT-PCR) amplification was performed using a StepOne™ Real-Time PCR System (Life Technologies, Foster City, CA, United States) using the next steps: 1) denaturation stage at 95 °C for 10 min, 2) followed by 50 cycles of denaturation at 92 °C for 15 s, 3) annealing/extension at 60 °C for 1 min, and 4) final extension stage of 30 s at 60 °C.

### Plasma biochemical parameters

Glucose and triglycerides levels were measured using commercial kits from Spinreact (glucose-HK, ref. 1001200; TAG, ref. 10013110) and following manufactures’ instructions. The intra-assay coefficients of variation were <1% and <0.4%, and inter-assay coefficients of variation were <1.5% and <3.6% for glucose and triglycerides, respectively. Absorbances were obtained using a BIO-TEK PowerWaveTM 340 microplate reader and the BIO-TEK KC JuniorTM program (Bio-Tek Instruments Inc., Winooski, VT, United States).

Plasma IL-6 TNF-α levels, as pro-oxidant cytokines, were determined by Immunology Multiplex Assay MILLIPLEX^®^ MAP Human High Sensitivity T Cell Magnetic Bead Panel 96-Well Plate Assay (HSTCMAG-28SK and HSCRMAG-32K, Merck Millipore, Darmstadt, Germany) and the Luminex^®^ 200TM System (Luminex Corp., Austin, TX, United States) according to the manufacturer’s instructions. The intra-assay coefficients of variation were <5%, and inter-assay coefficients of variation were <15%. Minimum detected value for IL-6 was 0.11 pg · ml^−1^, and for TNF-α was 0.16 pg · ml^−1^.

Also, plasma insulin and leptin levels were measured using MILLIPLEX^®^ MAP Human Metabolic Hormone Magnetic Bead Panel (HMHEMAG-34K, Millipore Sigma, Burlington, MA, United States) and Luminex^®^ 200TM System (Luminex Corp., Austin, TX, United States) according to the manufacturer’s instructions. The intra-assay coefficients of variation were <10%, and inter-assay coefficients of variation were <15%. Insulin data in pg · ml^−1^ was converted to mUI · L^−1^ (Knopp et al., 2019).

Based on glucose and insulin levels, some indexes were obtained. HOMA-IR (Homeostasis Model Assessment of Insulin Resistance) (Geloneze et al., 2009), and QUICKI (QUantitative Insulin sensitivity cheCK Index) (Sarafidis et al., 2007) were calculated as a previous study of the NutAF project described (Montes-de-Oca-García et al., 2021).

### Anthropometry and body composition

Height was registered in a standing position with a rod (SECA 225, range from 60 to 200 cm; 1 mm of precision). Waist circumference was measured using a plastic anthropometric tape (SECA 201; range from 0 to 205 cm; 1 mm of precision) at the midpoint. All measurements were registered by duplicated. Body mass (kg), body fat (kg), and lean body mass (kg) were evaluated through a multi-frequency bioelectrical bioimpedance analyzer with 8-point electrodes (TANITA-MC780MA, Barcelona, Spain). A specific posture was adopted by the participants according to the manufacturer’s instructions, moreover, the subjects were required to urinate before the test, to wear light clothes, and to remove any metal object, such as earrings, watches, etc. The BMI was calculated by dividing body mas in kilograms to squared height in meters.

### Basal metabolic rate

The Respiratory Exchange Ratio (RER) and resting fat oxidation were estimated in resting conditions through indirect calorimetry by using a Jaeger MasterScreen CPX^®^ (CareFusion, San Diego, CA, United States) gas analyzer, in a conditioned room (21 ± 1°C, 50 ± 2% relative humidity). The oxygen uptake (VO_2_) and carbon dioxide production (VCO_2_) were registered breath-by-breath and averaged every 20 s during 30 min, with the participant wearing a mask and lying in a supine position. A stable period of 5 min with a coefficient of variation for VO_2_ and VCO_2_ lower than 15% was selected for the analysis, these VO_2_ and VCO_2_ average values were then used to calculate the basal metabolism (kcal) by the Frayn’s equation (Frayn, 1983).

### Maximal fat oxidation and cardiorespiratory fitness

The MFO test consisted of a 3-min steps incremental test on a cycle ergometer (Lode Excalibur, Groningen, Netherlands) with 15 W increments in overweight/obese participants and 30 W in normal weight participants, maintaining a pedaling cadence of 60–80 rpm, until RER ≥1. After a 5-min recovery period, the VO_2_max test was performed (Achten et al., 2002), beginning at the load at which the MFO test ended. The VO_2_max test consisted of a 1-min incremental test until exhaustion, at the same load rate and cadence as described for the MFO test. A polynomial curve that best fits the present analysis was drawn for each subject by using the values of fat oxidation and % VO_2_max of each step. Specifically, the VO_2_ and VCO_2_ average values of the last 60 s of each step were used to estimate fat oxidation by indirect calorimetry using the mentioned Frayn’s equation (Frayn, 1983). Similarly, in order to calculate the % VO_2_max reached in each step, the VO_2_ average values were used. Then, MFO was expressed as absolute values and also relativized to legs lean mass divided by squared height (R-MFO).

### Statistical analysis

To verify the Hardy-Weinberg equilibrium and to describe both the genotypic and allelic frequencies, chi-squared test was applied. The Kolmogorov-Smirnov and Levene tests were used to check normality of distribution and homogeneity of variance, respectively.

All variables were separately analyzed and two CMR clusters were obtained. A CMR cluster according to sex was created with the standardized values (Z-score) [(value −mean)/standard deviation] of waist circumference, body fat percentage, SBP, DBP, blood glucose, and triglycerides. The second CMR cluster was created adding the TNF-α and IL-6 markers to the mentioned cluster model (Montes-de-Oca-García et al., 2020; Montes-de-Oca-García et al., 2021).

A one-way analysis of variance (ANOVA), followed by Bonferroni post hoc comparisons, was applied to determine the differences between the PPARGC1A gene rs8192678 C>T polymorphisms (CC, CT, and TT). In addition, a recessive model was included comparing through a Student *t*-test for independent samples the differences between both groups (CC vs. CT/TT). Differences between sexes were also assessed by using a Student *t*-test for independent samples. In order to determine the interaction of sex and the PPARGC1A gene rs8192678 C>T polymorphism, a two-way ANOVA and Bonferroni post hoc comparisons were carried out. In all group comparisons, the effect sizes were calculated, thus, statistical analysis included Cohen’s d for Student *t*-test for independent samples, eta squared (η^2^) for one-way ANOVA, and partial eta squared (η_p_^2^) for two-way ANOVA.

All analyses were performed by using the IBM SPSS Statistics 22 software (SPSS Inc., Chicago, IL, United States), with significance set at *p* < 0.05, and expressing data as mean ± standard deviation (SD).

## Results

Regarding the general characteristics of the participants that are shown in Table 1, statistically significant differences were found between sexes in height (higher in men; *p* < 0.001), lean body mass (higher in men; *p* < 0.001), body fat (higher in women; *p* = 0.010), VO_2_max per kilogram of body mass (higher in men; *p* < 0.001), basal metabolic rate (higher in men; *p* < 0.001), total energy expenditure (higher in men; *p* < 0.001), dietary energy intake (higher in men; *p* = 0.002), absolute MFO rate (higher in men; *p* = 0.016), R-MFO rate (higher in women; *p* = 0.040), SBP (higher in men; *p* = 0.002), fasting blood glucose (higher in men; *p* = 0.008), and leptin (higher in women; *p* < 0.001).

The Hardy-Weinberg equilibrium (HWE) test indicated that χ^2^ = 0.005, *p* = 0.945, suggesting that the population is consistent with HWE and confirming that the allele types were randomly sampled. The expected frequencies were CC (*p* = 0.4203) *n* = 31.1, CT (*p* = 0.4554) *n* = 33.7, and TT (*p* = 0.1230) *n* = 9.1. The distribution of PPARGC1A genotype frequencies was 42% for CC, 46% for CT, and 12% for TT.

The differences in health-related parameters between PPARGC1A gene variants (CC, CT, and TT) of the participants are presented in Table 2. In the one-way ANOVA, statistically significant differences were found in body composition. Specifically, there were significant differences in body mass (*p* = 0.002), BMI (*p* = 0.024), lean body mass (*p* = 0.024), body fat (*p* = 0.032), and waist circumference (*p* = 0.020). Moreover, statistically significant differences were found in the blood pressure recovery ratio (*p* < 0.001). No genotype * sex interactions were found in the two-way ANOVA for any of the analyzed variables (*p* > 0.050).

**TABLE 2:** Differences between the three PPARGC1A gene rs8192678 C>T polymorphisms in the analyzed variables.

	n	CC(*n* = 31)	CT(*n* = 34)	TT(*n* = 9)	*p*-value	η^2^
Number of Men/Women	—	18/13	21/13	7/2	—	—
Age (years)	74	21.4 ± 2.6	23.2 ± 4.4	24.2 ± 6.7	0.117	0.059
Height (cm)	74	170.8 ± 8.9	173.1 ± 8.8	173.1 ± 7.4	0.536	0.017
Body Mass (kg)	74	69.1 ± 10.4	80.8 ± 17.4	84.3 ± 16.8	**0.002** ^ab^	**0.157**
Body Mass Index (kg · m^−2^)	74	23.7 ± 4.1	27.1 ± 6.5	28.1 ± 4.8	**0.024** ^b^	**0.099**
Lean Body Mass (kg)	74	51.2 ± 7.2	56.2 ± 9.6	58.4 ± 7.5	**0.024** ^b^	**0.100**
Body fat (kg)	74	14.7 ± 8.6	21.2 ± 12.5	22.7 ± 11.2	**0.032** ^b^	**0.093**
Waist circumference (cm)	68	77.9 ± 11.5	86.2 ± 15.2	91.6 ± 16.9	**0.020** ^a^	**0.113**
VO_2_max (mL · kg^−1^ · min^−1^)	73	42.7 ± 11.3	39.4 ± 11.8	40.9 ± 14.2	0.545	0.017
Basal metabolism (kcal · min^−1^)	73	1.2 ± 0.2	1.3 ± 0.3	1.3 ± 0.2	0.130	0.057
Total energy expenditure (kcal/day)	67	2,291 ± 321	2,421 ± 489	2,498 ± 360	0.313	0.036
Dietary energy intake (kcal/day)	73	2,527 ± 640	2,423 ± 676	2,993 ± 758	0.084	0.068
Energy balance (kcal/day)	66	243 ± 624	−2 ± 738	495 ± 884	0.152	0.058
Resting Fat Oxidation (mg · min^−1^)	73	95 ± 24	101 ± 31	109 ± 29	0.398	0.026
Absolute MFO (mg · min^−1^)	73	359 ± 130	386 ± 170	388 ± 188	0.763	0.008
R-MFO (mg (kg · m ^−2^)^−1^ · min^−1^)	73	6.8 ± 2.7	7.1 ± 2.9	6.8 ± 3.3	0.951	0.001
Total MVPA (min/week)	67	391 ± 120	371 ± 152	311 ± 134	0.304	0.037
MD adherence (0–14 range)	73	6.9 ± 1.5	6.7 ± 1.8	7.6 ± 2.4	0.373	0.028
Systolic blood pressure (mmHg)	73	114.1 ± 11.4	114.7 ± 9.1	117.8 ± 10.9	0.627	0.013
Diastolic blood pressure (mmHg)	73	68.1 ± 10.9	69.7 ± 8.9	69.3 ± 9.3	0.788	0.007
Blood pressure recovery (ratio)	68	0.90 ± 0.05	0.91 ± 0.06	1.00 ± 0.08	**<0.001** ^a^	**0.234**
Fasting glucose (mg · dl^−1^)	74	103.0 ± 8.7	99.5 ± 11.3	99.8 ± 10.2	0.365	0.028
Fasting insulin (ng · ml^−1^)	74	0.8 ± 0.7	0.7 ± 0.4	1.1 ± 1.3	0.556	0.016
HOMA-IR index	74	5.7 ± 5.7	5.4 ± 3.4	7.8 ± 10.5	0.513	0.019
QUICKI index	74	0.31 ± 0.02	0.31 ± 0.02	0.30 ± 0.03	0.768	0.007
Fasting triglycerides (mg · dl^−1^)	74	73.4 ± 23.1	67.1 ± 26.0	68.2 ± 26.4	0.585	0.015
Tumor necrosis factor-α (ng · ml^−1^)	71	8.6 ± 5.8	7.7 ± 7.4	9.7 ± 1.3	0.778	0.007
Interleuking-6 (ng · ml^−1^)	55	0.5 ± 0.4	0.4 ± 0.2	0.3 ± 0.2	0.486	0.027
Leptin (ng · ml^−1^)	70	3.4 ± 4.4	4.1 ± 4.6	4.4 ± 4.5	0.758	0.008
Clustered CMR (Z-score)	67	0.03 ± 3.2	0.6 ± 3.2	1.3 ± 3.7	0.582	0.017
INFLAM-Clustered CMR (Z-score)	47	0.2 ± 2.8	0.2 ± 3.2	−0.3 ± 4.5	0.948	0.002

Values are presented as mean ± standard deviation. Genotype differences appear in bold (*p* < 0.05 in the one-way ANOVA). Abbreviations: MVPA, Moderate to vigorous physical activity; MD, Mediterranean diet; VO_2_max, Maximal oxygen uptake; R-MFO, Maximal fat oxidation rate relativized to legs lean mass divided by squared height; HOMA-IR, Homeostasis Model Assessment of Insulin Resistance; QUICKI, QUantitative Insulin sensitivity cheCK Index; CMR, Cardiometabolic risk; INFLAM-Clustered CMR, Cluster of CMR including IL-6 and TNF-α.

a Means significant differences between CC and TT

b between CT and TT.

c Between CC and CT in the Bonferroni post hoc comparisons.

The differences between groups in the recessive model (CC vs. CT/TT) are shown in Table 3, where the CC genotype showed lower age (*p* = 0.031), body mass (*p* < 0.001), BMI (*p* = 0.004), lean body mass (*p* = 0.008), body fat (*p* = 0.006), waist circumference (*p* = 0.006), basal metabolism (*p* < 0.001), and blood pressure recovery ratio (*p* = 0.047) than the CT/TT genotype group.

**TABLE 3:** Comparisons of the recessive model (CC vs. CT/TT) in the analyzed variables.

	*n*	CC(*n* = 31)	CT/TT(*n* = 43)	*p*-value	*d*
Number of Men/Women	—	18/13	28/15	—	—
Age (years)	74	21.4 ± 2.6	23.4 ± 4.9	**0.031**	−**0.50**
Height (cm)	74	170.8 ± 8.9	173.1 ± 8.4	0.263	−0.27
Body Mass (kg)	74	69.1 ± 10.4	81.5 ± 17.1	**<0.001**	−**0.88**
Body Mass Index (kg · m^−2^)	74	23.7 ± 4.1	27.2 ± 6.1	**0.004**	−**0.67**
Lean Body Mass (kg)	74	51.2 ± 7.2	56.6 ± 9.2	**0.008**	−**0.66**
Body fat (kg)	74	14.7 ± 8.6	21.5 ± 12.1	**0.006**	−**0.65**
Waist circumference (cm)	68	77.9 ± 11.4	87.3 ± 15.5	**0.006**	−**0.69**
VO_2_max (ml · kg^−1^ · min^−1^)	73	42.7 ± 11.3	39.7 ± 12.2	0.293	0.25
Basal metabolism (kcal · min^−1^)	73	1.2 ± 0.2	1.3 ± 0.2	**0.045**	−**0.50**
Total energy expenditure (kcal/day)	67	2,292 ± 321	2,439 ± 459	0.147	−0.37
Dietary energy intake (kcal/day)	73	2,527 ± 640	2,542 ± 724	0.923	−0.02
Energy balance (kcal/day)	66	244 ± 624	112 ± 790	0.477	0.18
Resting Fat Oxidation (mg · min^−1^)	73	96 ± 24	102 ± 30	0.289	−0.26
Absolute MFO (mg · min^−1^)	73	359 ± 130	387 ± 172	0.461	−0.18
R-MFO [mg (kg · m ^−2^)^−1^ · min^−1^]	73	6.8 ± 2.7	7.0 ± 2.9	0.824	−0.05
Total MVPA (min/week)	67	391 ± 120	357 ± 142	0.315	0.25
MD adherence (0–14 range)	73	6.9 ± 1.5	6.9 ± 1.9	0.987	0.01
Systolic blood pressure (mmHg)	73	114.1 ± 11.4	115.4 ± 9.4	0.578	−0.13
Diastolic blood pressure (mmHg)	73	68.1 ± 10.9	69.6 ± 8.9	0.494	−0.16
Blood pressure recovery (ratio)	68	0.90 ± 0.05	0.93 ± 0.10	**0.047**	−**0.49**
Fasting glucose (mg · dl^−1^)	74	103.0 ± 8.7	99.6 ± 11.0	0.155	0.35
Fasting insulin (ng · ml^−1^)	74	0.8 ± 0.7	0.8 ± 0.7	0.860	−0.04
HOMA-IR index	74	5.7 ± 5.7	5.9 ± 5.6	0.876	−0.04
QUICKI index	74	0.31 ± 0.02	0.30 ± 0.02	0.568^a^	0.39
Fasting triglycerides (mg · dl^−1^)	74	73.4 ± 23.1	67.3 ± 25.8	0.302	0.25
Tumor necrosis factor-α (ng · ml^−1^)	71	8.6 ± 5.8	8.1 ± 8.7	0.772	0.07
Interleuking-6 (ng · mL^−1^)	55	0.5 ± 0.4	0.4 ± 0.2	0.378	0.24
Leptin (ng · ml^−1^)	70	3.4 ± 4.4	4.2 ± 4.5	0.468	−0.18
Clustered CMR (Z-score)	67	0.03 ± 3.2	0.7 ± 3.3	0.373	−0.23
INFLAM-Clustered CMR (Z-score)	47	0.2 ± 2.8	0.1 ± 3.4	0.880	0.04

Values are presented as mean ± standard deviation. Genotype differences appear in bold (*p* < 0.05 in the Student t-test). Abbreviations: d, Cohen’s d; MVPA, Moderate to vigorous physical activity; MD, Mediterranean diet; VO_2_max, Maximal oxygen uptake; R-MFO, Maximal fat oxidation rate relativized to legs lean mass divided by squared height; HOMA-IR, Homeostasis Model Assessment of Insulin Resistance; QUICKI, QUantitative Insulin sensitivity cheCK Index; CMR, Cardiometabolic risk; INFLAM-Clustered CMR, Cluster of CMR including IL-6 and TNF-α.

a Indicates a genotype*sex interaction in the two-way ANOVA.

**FIGURE 1:**
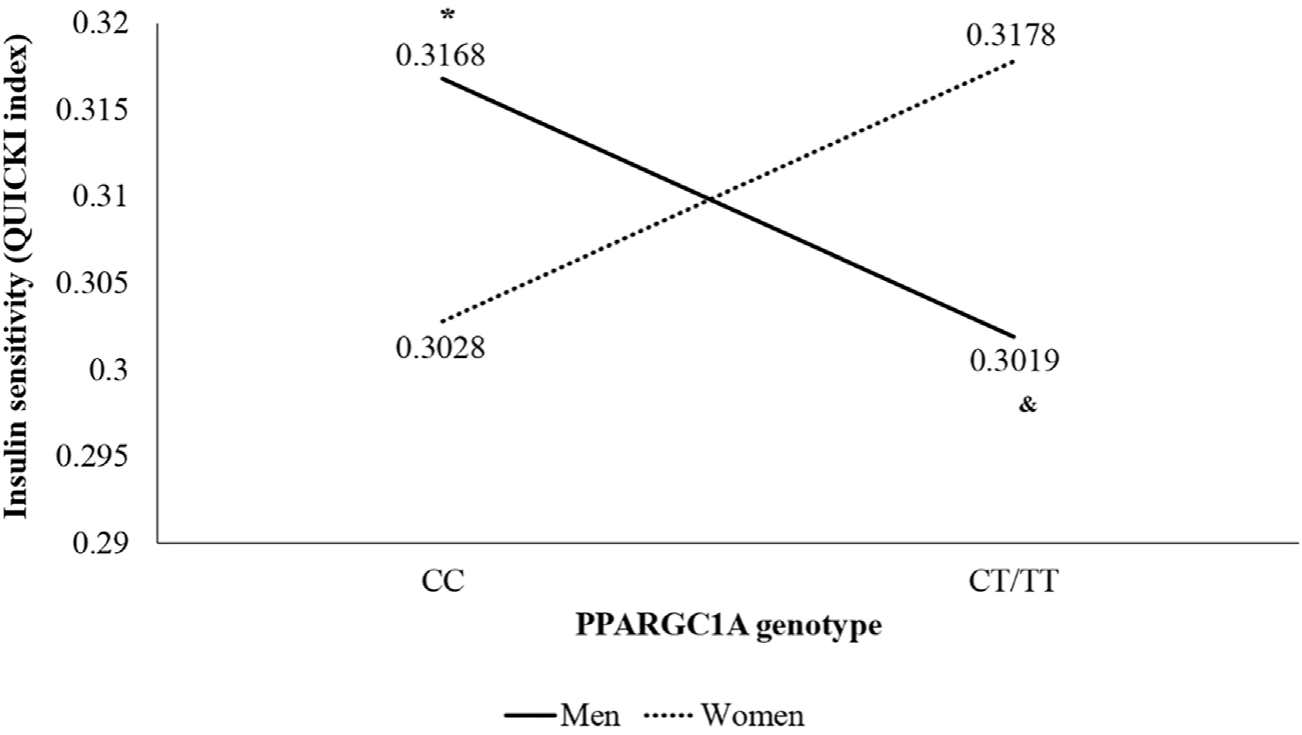
Interaction of sex and genotype of PPARGC1A gene rs8192678 C>T polymorphism in insulin sensitivity (QUICKI index) using the recessive model (CC vs. CT/TT). *p < 0.050 vs. other genotype groups. & *p* < 0.050 vs. women of the same genotype group.

Moreover, in the two-way ANOVA, a genotype * sex interaction was found in QUICKI (F (1,70) = 6.121; *p* = 0.016; η_p_^2^ = 0.080), presented in Figure 1, with statistically significant differences between CC and CT/TT in men (*p* = 0.049) and between men and women inside the CT/TT group (*p* = 0.049).

## Discussion

The main finding of the present cross-sectional study is that PPARGC1A gene rs8192678 C>T polymorphism is associated with body composition, basal metabolic rate, and insulin sensitivity (QUICKI index). In our study, the T allele carriers had higher values of body mass, BMI, lean body mass, body fat, and waist circumference. The influence of PPARGC1A gene rs8192678 C>T polymorphism on body composition is not explained by differences in cardiorespiratory fitness, age, height or MFO, since all groups showed similar values. Hence, our results showed that individuals with the PPARGC1A rs8192678 (T) genotype have a higher risk of developing obesity and metabolic disorders such as insulin resistance, according to previous data (Yang et al., 2011).

In addition, specific-gender analyses are encouraged in the literature; in this line, a main result in our study showed a genotype*sex interaction in QUICKI index, with lower values of QUICKI index (which means a lower insulin sensitivity) in T allele carriers in men and with higher values of QUICKI index (which means a higher insulin sensitivity) in women inside the CT/TT group. In agreement, epidemiological studies revealed gender differences regarding the association of rs8192678 with the metabolic syndrome pathology and insulin resistance (Csép et al., 2017). The insulin sensitivity finding in men is partially contradicted by body composition results, since the amount of muscle mass is directly associated with insulin sensitivity in young overweight adults (Haines et al., 2020) and, in our study, the T allele carriers had higher levels of lean body mass. However, in addition to having a greater amount of muscle mass, the T allele carriers also had higher levels of fat mass, BMI and waist circumference than C allele carriers, which would explain a lower insulin sensitivity, since adiposity is directly associated with decreased insulin sensitivity (Smith et al., 2020). Otherwise, the higher score in the QUICKI index in women could be explained by the higher levels of estrogens, which confers an increased insulin sensitivity and, therefore, a cardioprotective effect compared with men (Goossens et al., 2021).

In connection with biochemical parameters, the scientific evidence on insulin and glucose agrees with the results of our study. In fact, in the study of Mirzaei et al. (2012) there were no statistically significant differences in the levels of fasting blood glucose and insulin between the three PPARGC1A gene rs8192678 C>T polymorphism variants. In addition, the association between PPARGC1A gene rs8192678 C>T polymorphism and CMR has been slightly studied through individual cardiovascular risk factors such as body composition (Mirzaei et al., 2012; Franks et al., 2014; Zamaninour et al., 2018), type 2 diabetes-related parameters (Zhu et al., 2017), inflammatory markers and lipid profile (Mirzaei et al., 2012), and blood pressure (Vimaleswaran et al., 2008). Therefore, to our knowledge, this is the first study in which the relationship between PPARGC1A gene rs8192678 C>T polymorphism and a cluster of CMR factors is investigated. However, we did not find any association with this polymorphism.

Regarding blood pressure, it has been suggested that PPARGC1A gene rs8192678 (Gly482Ser) polymorphism is associated with blood pressure in young adults, such that Ser482 allele homozygotes have higher values of blood pressure than Gly482 allele homozygotes (Vimaleswaran et al., 2008). Similarly, in our study it was shown that those subjects carrying the T allele (Ser482) have a worse recovery of systolic blood pressure after exercise, and, consequently, they also have a higher cardiovascular risk (Michaelides et al., 2013). Thus, our study is the first to analyze the differences in the recovery of SBP depending on the three PPARGC1A gene rs8192678 C>T polymorphism variants. In fact, our results offer a very useful tool to detect cardiometabolic risk early and from a genetic perspective.

The findings of our study confirm some of the results of previous studies on the influence of PPARGC1A gene rs8192678 C>T polymorphism on health-related parameters. For example, Zamaninour et al. (2018) demonstrated that PPARGC1A gene rs8192678 C>T polymorphism is related to obesity onset age in obese adults without acute or chronic diseases. However, despite the association of adiposity and chronic inflammation, the study of Mirzaei et al. (2012) showed no statistically significant differences in the levels of C-reactive protein and fasting blood triglycerides between the three PPARGC1A gene rs8192678 C>T polymorphism variants, so the results of our study are consistent since no significant differences were found in TNF-α, IL-6 or fasting plasma triglycerides. Besides, contrary to our data, it has been observed that the 482Ser of PPARGC1A is associated with lower leptin levels and lower cardiometabolic risk factors despite obesity in Mexican-Mestizos (Vázquez-Del Mercado et al., 2015).

Moreover, higher values of basal metabolic rate and total energy expenditure were observed in the T allele carriers, that was accompanied by an increased adiposity was observed in this group. This controversial data could be explained because the basal metabolic rate and energy expenditure are directly related to lean body mass, in addition to other factors such as aging and chronic diseases (Zampino et al., 2020). The observed higher lean mass in the PPARGC1A rs8192678 (T) genotype is likely due to the increased fat mass, since, after 100 days of overfeeding, lean mass has been shown to increase approximately 3 kg with a fat mass accumulation of approximately 5 kg in a group of young adults (Ernersson et al., 2010), as in our study. Likewise, the higher levels of adiposity of the T allele carriers could justify the worse results of the blood pressure recovery ratio. In point of fact, BMI and waist circumference have previously been negatively associated with blood pressure recovery in healthy adults (Dimkpa and Ugwu, 2010).

Previously, PPARGC1A gene rs8192678 polymorphism has been associated with a subcutaneous adiposity accumulation and with insulin resistance in obese adults (Franks et al., 2014). In addition, Mirzaei et al. (2012) investigated the possible correlation with obesity-related conditions and resting energy expenditure in healthy adults with different states of body mass, finding significant differences in BMI, fat mass, insulin levels, and resting energy expenditure among the three genotypes of PPARGC1A gene rs17574213 polymorphism (CC, CT, and TT). Specifically, this study reported statistically significant differences between the three PPARGC1A gene rs8192678 C>T polymorphism variants in the QUICKI index, with a general tendency towards a worse prognosis in those subjects with the T allele compared to the C allele. However, this study showed no significant differences in HOMA-IR and basal metabolic rate.

Nevertheless, even though significant differences were found in our study in basal metabolism, no significant differences were found in MFO. This lack of significance could be explained by the absence of significant differences in VO_2_max since it has been shown that MFO depends directly on VO_2_max (Randell et al., 2017). These results indicate that the PPARGC1A gene rs8192678 C>T polymorphism is associated with basal metabolism (Muller et al., 2003), but not with fat metabolism during exercise, which is influenced by other factors such as age, sex, diet, and physical fitness level (Compher et al., 2006). Hence, the observed increase of adiposity in the T allele in our study could be mainly due to the activation of adipogenesis (de sá et al., 2017; Stachowiak et al., 2018), since this group showed a normal fat oxidation capacity and increased basal metabolic rate compared to C allele carriers.

Regarding VO_2_max, our findings confirm the results of previous studies in which no statistically significant differences were found in VO_2_max. For example, He et al. (2008) showed that VO_2_max was not associated with the rs8192678G>A polymorphism in Chinese healthy young adults. On the contrary, another study reported that in untrained men the G allele carriers at rs8192678 typically have higher VO_2_max values (Ring-Dimitriou et al., 2014). Thus, it becomes evident that there is controversy regarding this issue. Nevertheless, a relatively recent systematic review (Petr et al., 2018) reported that the aerobic training response is significantly influenced by PPAR’s gene polymorphisms and their coactivators. Indeed, in some genetically predisposed individuals, the aerobic training can negatively influence glucose metabolism and VO_2_max (Petr et al., 2018). Likewise, it has been shown that the SNP Gly482Ser in the PPARGC1A gene impairs exercise-induced slow-twitch muscle fiber transformation in humans (Steinbacher et al., 2015). Thus, more studies are also needed in this regard.

Concerning physical activity, our results contradict other studies in which significant differences were found in physical activity levels. For instance, a twin study of Gielen et al. (2014) showed that AA carriers in PPARGC1A gene rs8192678G>A polymorphism presented higher physical activity levels than GG carriers. Besides, another study reported that in healthy Korean children the genetic effects of the PGC-1α genotypes on health-related parameters might be modulated by lifestyle factors such as physical activity (Ha et al., 2015). Hence, more studies about this topic are also needed.

Respecting energy balance, Muller et al. (2003) showed that individuals with Gly/Gly showed a lower rate of fat oxidation than individuals with either Gly/Ser or Ser/Ser, resulting in a more positive energy balance in Gly/Gly carriers compared with in Gly/Ser or Ser/Ser carriers. Despite not finding significant differences in our study, the same happened, so an increased energy balance due to reduced fat oxidation would predict an increased cardiometabolic risk. Otherwise, we have not found any study that relates the PPARGC1A gene rs8192678 C>T polymorphism with the Mediterranean diet adherence. Specifically, in our study no significant association was found in this regard, which seems to indicate that the PPARGC1A gene rs8192678 C>T polymorphism is not associated with the Mediterranean diet adherence.

It should be noted that this study is not without limitations. We are aware that transient occasion factors may bias variable measurements and serve as sources of common variance which is a major limitation of the design of cross-sectional studies. Moreover, the number of participants included in the present study is scarce to make solid conclusions (e.g., there were only nine participants of which only two were women in the TT group). All our subjects were young and healthy (despite overweight/obesity), living in the province of Cadiz (south of Spain), thus a multicenter study is encouraged to compare our results with other populations. Furthermore, participants were excluded if they are smokers or have any cardiovascular disease, among others, potentially compromising external validity of results. Therefore, future studies should include a larger cohort with longitudinal design, also prioritizing results from genome-wide association studies. Regarding the body composition assessment, Dual Energy X-ray Absorptiometry (DXA) can be included to obtain a more precise evaluation instead of bioimpedance.

However, a strength of this study is the assessment of MFO and VO_2_max by using an exercise protocol test and adequate equipment under laboratory-controlled conditions. Moreover, the number of registered outcomes, including biochemical parameters, brings strengths to the design. Likewise, this study provides novel results, since to our knowledge no study to date has investigated the relationship between PPARGC1A gene rs8192678 C>T polymorphism and health-related parameters such as blood pressure recovery ratio, the ability to oxidize fat during exercise, diet, and clustered CMR factors.

## Conclusion

In conclusion, PPARGC1A gene rs8192678 C>T polymorphism is associated with body composition, basal metabolism, and systolic blood pressure recovery ratio, with higher values in CT/TT groups. However, this polymorphism has little or no influence on VO_2_max, resting fat oxidation, MFO, physical activity, diet, systolic and diastolic blood pressure, biochemical parameters, and CMR. Moreover, our study highlighted sexual dimorphism in the influence of PPARGC1A gene rs8192678 C>T polymorphism on the QUICKI index, with a tendency towards a worse prognosis in those subjects with the T allele and male gender. Nonetheless, more studies are required to clarify the association between PPARGC1A gene rs8192678 C>T polymorphism and health-related parameters in a larger population.

## Data Availability

The original contributions presented in the study are included in the article/supplementary materials, further inquiries can be directed to the corresponding author.
